# Influenza Activity — United States, 2013–14 Season and Composition of the 2014–15 Influenza Vaccines

**Published:** 2014-06-06

**Authors:** Scott Epperson, Lenee Blanton, Krista Kniss, Desiree Mustaquim, Craig Steffens, Teresa Wallis, Rosaline Dhara, Michelle Leon, Alejandro Perez, Sandra S. Chaves, Anwar Abd Elal, Larisa Gubareva, Xiyan Xu, Julie Villanueva, Joseph Bresee, Nancy Cox, Lyn Finelli, Lynnette Brammer

**Affiliations:** 1 Influenza Division, National Center for Immunization and Respiratory Diseases, CDC

During the 2013–14 influenza season in the United States, influenza activity* increased through November and December before peaking in late December. Influenza A (H1N1)pdm09 (pH1N1) viruses predominated overall, but influenza B viruses and, to a lesser extent, influenza A (H3N2) viruses also were reported in the United States. This influenza season was the first since the 2009 pH1N1 pandemic in which pH1N1 viruses predominated and was characterized overall by lower levels of outpatient illness and mortality than influenza A (H3N2)–predominant seasons, but higher rates of hospitalization among adults aged 50–64 years compared with recent years. This report summarizes influenza activity in the United States for the 2013–14 influenza season (September 29, 2013–May 17, 2014†) and reports recommendations for the components of the 2014–15 Northern Hemisphere influenza vaccines.

## Viral Surveillance

**FIGURE 1:**
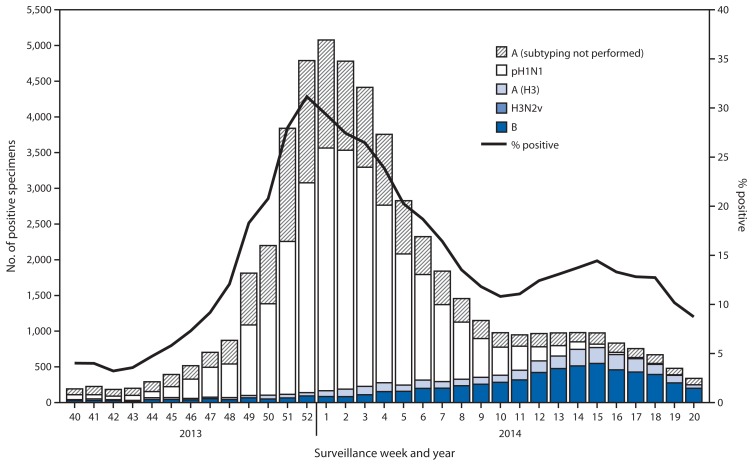
Number* and percentage of respiratory specimens testing positive for influenza, by type, subtype, surveillance week, and year — World Health Organization and National Respiratory and Enteric Virus Surveillance System collaborating laboratories, United States, 2013–14 influenza season^†^ ^*^ N = 53,470. ^†^ Data reported as of May 30, 2014.

During September 29, 2013–May 17, 2014, World Health Organization and National Respiratory and Enteric Virus Surveillance System collaborating laboratories in the United States tested 308,741 specimens for influenza viruses; 53,470 (17.3%) were positive (Figure 1). Of the positive specimens, 46,727 (87.4%) were influenza A viruses, and 6,743 (12.6%) were influenza B viruses. Among the seasonal influenza A viruses, 31,353 (67.1%) were subtyped; 28,323 (90.3%) were pH1N1 viruses, and 3,030 (9.7%) were influenza A (H3) viruses. In addition, one variant influenza A (H3N2)§ virus (H3N2v) was identified.

During the 2013–14 season, pH1N1 viruses were the predominant viruses in circulation nationally, with fewer influenza B viruses and influenza A (H3) viruses also identified. Using the percentage of specimens testing positive for influenza to determine the peak of influenza activity, the peak occurred during surveillance week 52 (the week ending December 28, 2013) nationally; however, differences among the 10 U.S. Department of Health and Human Services regions¶ in the timing of influenza activity were observed. Activity in Region 4 in the southern United States peaked earliest, during the week ending December 7, 2013 (surveillance week 49), and activity in Regions 2 and 3 in the eastern United States peaked latest, during the week ending January 25, 2014 (week 4).

Whereas pH1N1 activity peaked between late December and late January, influenza B activity occurred later in the influenza season. Influenza A viruses predominated until late March, and influenza B viruses became the most commonly identified virus nationally during the week ending March 29, 2014 (week 13). The intensity and timing of influenza B activity varied geographically. One region (Region 4) never reported a single week during which influenza B viruses predominated, whereas influenza B viruses were predominant in Region 6 from week 9 (the week ending March 1, 2014) through week 20 (the week ending May 17, 2014). During the late season increase in influenza B activity, the overall number of influenza A viruses decreased; however, the proportion of influenza A viruses subtyped as (H3) increased. In week 13 (the week ending March 29, 2014) influenza A (H3) viruses became the predominant influenza A virus nationally. Region 2 was most heavily impacted with late season influenza B activity, whereas Region 1 in the northeastern United States reported most late season influenza A (H3) activity. Region 2 identified 21.4% of all influenza B viruses nationally; Region 1 identified 21.4% of all influenza A viruses subtyped as (H3) nationally.

## Novel Influenza A Viruses

During the 2013–14 influenza season, one case of human infection with an H3N2v virus occurred during week 40 (the week ending October 5, 2013) in a child from Iowa with known direct exposure to swine. The child fully recovered, and no additional cases were identified in family members or other close contacts.

## Antigenic Characterization of Influenza Viruses

CDC antigenically characterized 2,905 influenza viruses collected and submitted by U.S. laboratories since October 1, 2013, including 2,036 pH1N1 viruses, 426 influenza A (H3N2) viruses, and 443 influenza B viruses. Of the 2,036 pH1N1 viruses tested, 2,033 (99.9%) were antigenically similar to A/California/7/2009, the influenza A (H1N1) component of the 2013–14 Northern Hemisphere influenza vaccines. Three viruses (0.1%) of the 2,036 tested showed reduced titers with ferret antiserum raised against A/California/7/2009. Of the 426 influenza A (H3N2) viruses tested, 406 (95.3%) were antigenically similar to A/Texas/50/2012, the influenza A (H3N2) component of the 2013–14 Northern Hemisphere vaccines. Twenty (4.7%) of the 426 tested showed reduced titers with antiserum produced against A/Texas/50/2012.

Of the 443 influenza B viruses tested, 323 (72.9%) belonged to the B/Yamagata lineage, and 322 (99.7%) were antigenically similar to B/Massachusetts/2/2012, the influenza B component of the 2013–14 Northern Hemisphere trivalent and quadrivalent influenza vaccines. One (0.3%) virus showed reduced titers with antiserum produced against B/Massachusetts/2/2012. The remaining 120 (27.1%) influenza B viruses belonged to the B/Victoria lineage and were antigenically similar to B/Brisbane/60/2008, the influenza B component of the 2013–14 Northern Hemisphere quadrivalent influenza vaccine.

## Resistance to Influenza Antiviral Medications

Since October 1, 2013, a total of 6,294 influenza virus specimens have been tested for resistance to influenza antiviral medications. All 508 influenza B viruses and 683 influenza A (H3N2) viruses tested were sensitive to both oseltamivir and zanamivir. Among the 5,103 pH1N1 viruses tested for resistance to oseltamivir, 59 (1.2%) were resistant, and all of the 1,890 viruses tested for resistance to zanamivir, including the 59 oseltamivir-resistant viruses, were sensitive. Resistance to the adamantanes (amantadine and rimantadine) persisted among influenza A viruses currently circulating globally (the adamantanes are not effective against influenza B viruses).

## Composition of the 2014–15 Influenza Vaccines

The Food and Drug Administration’s Vaccines and Related Biological Products Advisory Committee has determined that the 2014–15 influenza vaccines used in the United States have the same antigenic composition as those used in 2013–14. The trivalent vaccines should contain an A/California/7/2009-like (2009 H1N1) virus, an A/Texas/50/2012-like (H3N2) virus, and a B/Massachusetts/2/2012-like (B/Yamagata lineage) virus. The committee also recommended that quadrivalent vaccines contain a B/Brisbane/60/2008-like (B/Victoria lineage) virus (1). These recommendations were based on global influenza virus surveillance data related to epidemiology, antigenic and genetic characteristics, serologic responses to 2013–14 seasonal vaccines, and the availability of candidate vaccine viruses and reagents.

## Outpatient Illness Surveillance

**FIGURE 2:**
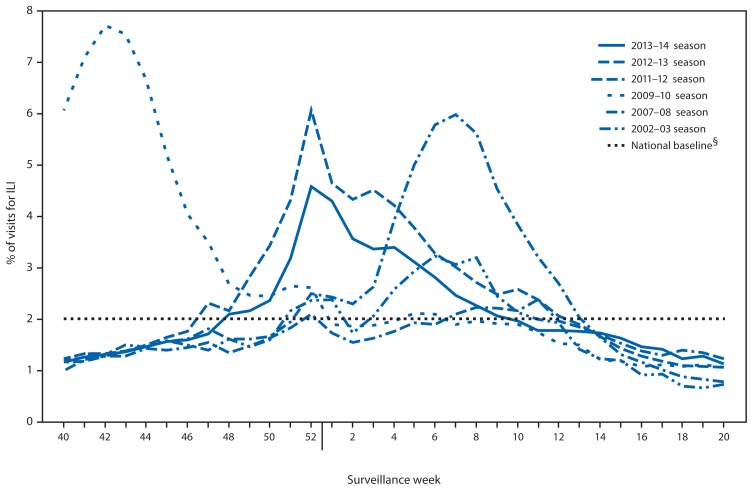
Percentage of visits for influenza-like illness (ILI)* reported to CDC, by surveillance week — Outpatient Influenza-Like Illness Surveillance Network, United States, 2013–14 influenza season and selected previous seasons^†^ * Defined as a fever of ≥100.0°F (≥37.8°C), oral or equivalent, and cough or sore throat, in the absence of a known cause other than influenza. ^†^ Data as of May 30, 2014. ^§^ The national baseline is the mean percentage of visits for ILI during weeks with little or no influenza virus circulation (noninfluenza periods) for the previous three seasons plus two standard deviations. A noninfluenza period is defined as ≥2 consecutive weeks in which each week accounted for <2% of the season’s total number of specimens that tested positive for influenza. Use of the national baseline for regional data is not appropriate.

Nationally, the weekly percentage of outpatient visits for ILI** to health-care providers participating in the U.S. Outpatient Influenza-Like Illness Surveillance Network (ILINet) was at or above the national baseline level†† of 2.0% for 15 consecutive weeks during the 2013–14 influenza season (Figure 2). The peak percentage of outpatient visits for ILI was 4.6%, and occurred in the week ending December 28, 2013 (week 52). During the 2012–13 influenza season, when influenza A (H3N2) virus was the predominant circulating virus, the peak percentage of outpatient visits for ILI was 6.1% and also occurred in late December. During the 2013–14 season, on a regional level, the percentage of visits for ILI exceeded region-specific baselines in all 10 regions for 8 consecutive weeks (Regions 7 and 10) and 22 consecutive weeks (Region 1).

What is already known on this topic?

CDC collects, compiles, and analyzes data on influenza activity year-round in the United States. Substantial influenza activity generally begins in the fall and continues through the winter and spring months; however, the timing and severity of influenza activity varies by geographic location and season.

What is added by this report?

The 2013–14 influenza season was the first influenza A (H1N1)pdm09–predominant season since the emergence of the virus in 2009, and also had later-season influenza B activity. The highest hospitalization rates were among adults aged ≥65 years, which is consistent with previous influenza seasons; hospitalization rates among those aged 50 to 64 years were significantly higher than in all years since the 2009 pandemic. Nearly all of the influenza virus specimens sent to CDC for antigenic characterization were similar to the components of the 2013–14 Northern Hemisphere influenza vaccine. The Food and Drug Administration has recommended that the 2014–15 influenza vaccines used in the United States have the same antigenic composition as those used in 2013–14.

What are the implications for public health practice?

Influenza surveillance, including for novel influenza viruses, should continue throughout the summer months, and health-care providers should consider influenza as a cause of respiratory illness even outside the typical season. Although influenza viruses typically circulate at low levels during the summer months, timely empiric antiviral treatment is recommended for patients with severe, complicated, or progressive influenza illness and those at higher risk for influenza complications; treatment can be considered for others if it can be started within 48 hours of illness onset.

ILINet data are used to produce a weekly jurisdiction-level measure of ILI activity,§§ ranging from minimal to high. The number of jurisdictions experiencing elevated ILI activity peaked during the week ending December 28, 2013 (week 52), when 22 jurisdictions experienced high ILI activity. During recent previous seasons, the peak number of jurisdictions experiencing high ILI activity has ranged from eight (2008–09 season) to 44 (2009–10 season) in a given week.

## Geographic Spread of Influenza Activity

State and territorial epidemiologists determine the geographic distribution of influenza in their jurisdictions using all available data sources through a weekly influenza activity code.¶¶ The geographic distribution of influenza activity was most extensive during the week ending January 18, 2014 (week 3), when 41 states reported widespread influenza activity and nine states reported regional influenza activity. The number of jurisdictions reporting widespread or regional activity during the peak week of activity has ranged from 40 to 51 jurisdictions during the previous four influenza seasons.

## Influenza-Associated Hospitalizations

**FIGURE 3:**
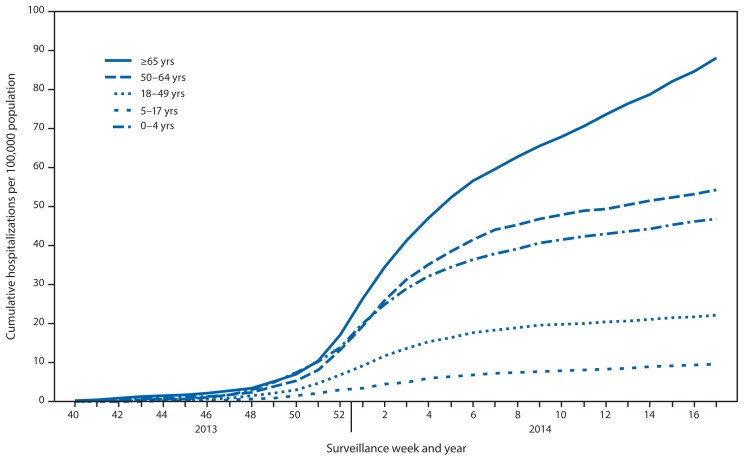
Cumulative rates of hospitalization for laboratory-confirmed influenza, by age group and surveillance week and year — FluSurv-NET* surveillance system, United States, 2013–14 influenza season^†^ ^*^ FluSurv-NET conducts population-based surveillance for laboratory-confirmed influenza-associated hospitalizations in children aged <18 years (since the 2003–04 influenza season) and adults aged ≥18 years (since the 2005–06 influenza season). FluSurv-NET covers approximately 70 counties in the 10 Emerging Infections Program states (California, Colorado, Connecticut, Georgia, Maryland, Minnesota, New Mexico, New York, Oregon, and Tennessee) and additional Influenza Hospitalization Surveillance Project states (Michigan, Ohio, and Utah). ^†^ Data as of May 30, 2014.

CDC monitors hospitalizations associated with laboratory-confirmed influenza virus infections using the Influenza Hospitalization Surveillance Network (FluSurv-NET).*** Cumulative hospitalization rates (per 100,000 population)††† were calculated by age group based on 9,635 reported influenza hospitalizations during October 1, 2013–April 30, 2014. Among 9,586 cases with influenza type specified, 8,497 (88.2%) were associated with influenza A virus infection, 1,046 (10.9%) with influenza B virus infection, and 43 (0.4%) were associated with mixed influenza A and influenza B virus infections. Persons aged 18–64 years accounted for 57.4% of reported hospitalizations. The cumulative incidence for all age groups for the period October 1, 2013–April 30, 2014, was 35.6 per 100,000 (Figure 3). The cumulative hospitalization rate (per 100,000 population) by age group for this period was 46.9 (for 0–4 years), 9.5 (5–17 years), 22.0 (18–49 years), 54.3 (50–64 years), and 88.1 (≥65 years). During the past four influenza seasons, age-specific hospitalization rates have ranged from 15.9 to 77.4 (0–4 years), 4.0 to 27.2 (5–17 years), 4.2 to 23.4 (18–49 years), 8.1 to 40.6 (50–64 years), and 25.7 to 183.1 (≥65 years).

As of May 30, 2014, among the FluSurv-NET adult patients for whom medical chart data were available, 89.0% had at least one underlying medical condition. The most frequent underlying medical conditions identified were obesity (42.9%), metabolic disorders (36.0%), and cardiovascular disease (34.6%). Among children hospitalized with laboratory-confirmed influenza and for whom medical chart data were available, 57.0% had at least one underlying medical condition. The most commonly identified conditions were asthma (25.4%) and neurologic disorders (14.1%). Among the 882 hospitalized women of childbearing age (15–44 years), 197 (22.3%) were pregnant.

## Pneumonia and Influenza-Associated Mortality

**FIGURE 4:**
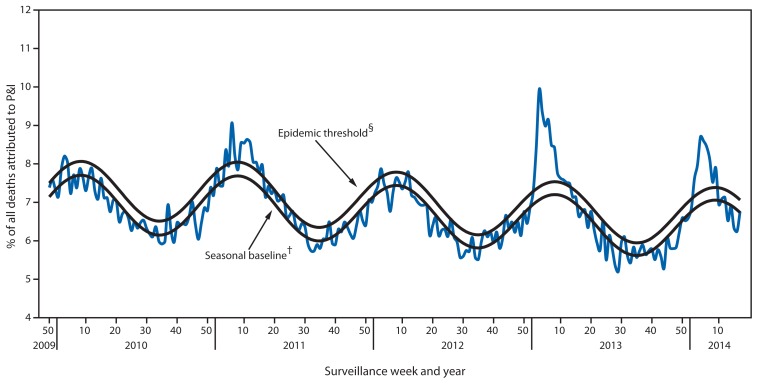
Percentage of all deaths attributable to pneumonia and influenza (P&I), by surveillance week and year — 122 Cities Mortality Reporting System, United States, 2009–2014* ^*^ Data as of May 30, 2014. ^†^ The seasonal baseline proportion of P&I deaths is projected using a robust regression procedure, in which a periodic regression model is applied to the observed percentage of deaths from P&I reported by the 122 Cities Mortality Reporting System during the preceding 5 years. ^§^ The epidemic threshold is set at 1.645 standard deviations above the seasonal baseline.

During the 2013–14 influenza season, the percentage of deaths attributed to pneumonia and influenza (P&I) exceeded the epidemic threshold§§§ for 8 consecutive weeks, from January 11, 2014 to March 1, 2014 (weeks 2–9). The percentage of deaths attributed to P&I peaked at 8.7% during the week ending January 25, 2014 (week 4) (Figure 4). From the 2008–09 influenza season through the 2012–13 season, the peak percentage of P&I deaths has ranged from 7.9% to 9.9%, and the total number of consecutive weeks at or above the epidemic threshold has ranged from 1 to 13.

## Influenza-Associated Pediatric Mortality

For the 2013–14 influenza season, 96 laboratory-confirmed, influenza-associated pediatric deaths were reported from 30 states, New York City, and Chicago. The deaths included 18 children aged <6 months, 24 aged 6–23 months, eight aged 2–4 years, 27 aged 5–11 years, and 19 aged 12–17 years; mean and median ages were 6.0 years and 4.6 years, respectively. Among the 96 deaths, 79 deaths were associated with influenza A virus infections (43 with pH1N1 viruses, two with an A [H3] virus, and 34 with influenza A viruses for which subtyping was not performed), 13 deaths were associated with influenza B viruses, two deaths were associated with an influenza virus for which the type was not determined, and two deaths were associated with an influenza A and influenza B virus coinfection. Of 90 children with known medical history, 49 (54.4%) had at least one high-risk medical condition. Neurologic disorders (29 [32.2%]) and pulmonary disease (17 [18.9%]) were the most commonly identified conditions.

Since influenza-associated pediatric mortality became a nationally notifiable condition in 2004, the total number of influenza-associated pediatric deaths has ranged from 35 to 171 per season; this excludes the 2009 pandemic, when 348 pediatric deaths were reported to CDC during April 15, 2009–October 2, 2010.

### Discussion

The 2013–14 influenza season peaked in late December with pH1N1 viruses predominating nationally and in all 10 regions. Activity decreased through January and February, but a late season increase in influenza B activity occurred in March, and influenza B viruses became the predominant virus nationally in week 13 (the week ending March 29, 2014). Nearly all of the influenza virus specimens sent to CDC for further antigenic characterization were similar to the components of the 2013–14 Northern Hemisphere vaccines.

After several recent influenza A (H3N2)–predominant seasons, 2013–14 was the first pH1N1–predominant season since the 2009 pH1N1 pandemic. During the 2009 pandemic, adults aged 50–64 years had the highest mortality rate and second highest influenza-associated hospitalization rate, and during the 2013–14 season, adults again were at high risk of severe influenza illness. The cumulative incidence of hospitalization among adults aged 50–64 years during the 2013–14 season was well above the range of rates seen in seasons following the pandemic, whereas hospitalization rates in all other age groups were within the range seen in recent years. This age distribution of hospitalizations is likely attributable to several factors, including lack of cross-protective immunity to pH1N1 and lower influenza vaccination coverage among persons in this age group (2).

Testing for seasonal influenza viruses and monitoring for novel influenza A virus infections should continue year-round, as should specimen submission to CDC for further antigenic and genetic analysis and antiviral resistance monitoring. Human infections with novel influenza A viruses were identified in greater numbers during the summer months of 2012 and 2013 (3,4) and might also occur during the summer months of 2014. An H3N2v virus that had acquired the matrix (M) gene from pH1N1 was first identified in pigs in 2010, and after being identified in 12 human patients in 2011 became the most commonly identified novel influenza A virus in the United States. Cases were most often associated with prolonged direct contact with swine in agricultural fair settings (3). Limited human-to-human spread of this virus has been detected, but no sustained community spread of H3N2v has been identified. The larger H3N2v outbreaks in 2012 and 2013 in the United States and continued identification of influenza A (H7N9) viruses (5) and other avian influenza viruses in humans outside the United States highlight the importance of ongoing monitoring for novel influenza A viruses throughout the year.

Although influenza activity in summer in the United States typically is low, cases of influenza, and even influenza outbreaks, are detected in the United States throughout the summer. Health-care providers should remain vigilant and consider influenza as a potential cause of summer respiratory illnesses, and also consider treatment with influenza antiviral medications for those at high risk for influenza-associated complications, as recommended by the Advisory Committee on Immunization Practices (6). Health-care providers also should consider novel influenza virus infections in persons with ILI and swine exposure, and those with severe acute respiratory infection after travel to areas where those viruses have been identified previously. Public health laboratories should immediately send to CDC any specimens that cannot be typed or subtyped using standard methods and submit all specimens that are otherwise unusual, including all summer specimens, as soon as possible after identification.

Influenza surveillance reports for the United States are posted online at CDC weekly and are available at http://www.cdc.gov/flu/weekly. Additional information regarding influenza viruses, influenza surveillance, influenza vaccine, influenza antiviral medications, and novel influenza A virus infections in humans is available at http://www.cdc.gov/flu.

## Footnotes

* The CDC influenza surveillance system collects information in five categories from eight data sources: 1) viral surveillance (World Health Organization collaborating laboratories, the National Respiratory and Enteric Virus Surveillance System, and novel influenza A virus case reporting); 2) outpatient illness surveillance (U.S. Outpatient Influenza-like Illness Surveillance Network); 3) mortality (122 Cities Mortality Reporting System and influenza-associated pediatric mortality reports); 4) hospitalizations (Influenza Hospitalization Surveillance Network [FluSurv-NET], which includes the Emerging Infections Program and Influenza Hospitalization Surveillance Project); and 5) summary of the geographic spread of influenza (state and territorial epidemiologist reports).

† Data as of May 30, 2014.

§ Influenza viruses that normally circulate in pigs are called “variant” viruses when they are found in humans. Influenza A (H3N2) variant viruses (H3N2v) with the matrix (M) gene from the pH1N1 pandemic virus were first detected in humans in July 2011. Cases of H3N2v infection have been confirmed in humans, mostly associated with prolonged exposure to pigs at agricultural fairs.

¶ *Region 1:* Connecticut, Maine, Massachusetts, New Hampshire, Rhode Island, and Vermont. *Region 2:* New Jersey, New York, Puerto Rico, and the U.S. Virgin Islands. *Region 3:* Delaware, the District of Columbia, Maryland, Pennsylvania, Virginia, and West Virginia. *Region 4:* Alabama, Florida, Georgia, Kentucky, Mississippi, North Carolina, South Carolina, and Tennessee. *Region 5:* Illinois, Indiana, Michigan, Minnesota, Ohio, and Wisconsin. *Region 6:* Arkansas, Louisiana, New Mexico, Oklahoma, and Texas. *Region 7:* Iowa, Kansas, Missouri, and Nebraska. *Region 8:* Colorado, Montana, North Dakota, South Dakota, Utah, and Wyoming. *Region 9:* Arizona, California, Hawaii, Nevada, American Samoa, Commonwealth of the Northern Mariana Islands, Federated States of Micronesia, Guam, Marshall Islands, and Republic of Palau. *Region 10:* Alaska, Idaho, Oregon, and Washington.

** Defined as a temperature of ≥100.0°F (≥37.8°C), oral or equivalent, and cough or sore throat, in the absence of a known cause other than influenza.

†† The national and regional baselines are the mean percentage of visits for ILI during weeks with little or no influenza virus circulation (noninfluenza periods) for the previous three seasons plus two standard deviations. A noninfluenza period is defined as ≥2 consecutive weeks in which each week accounted for <2% of the season’s total number of specimens that tested positive for influenza. National and regional percentages of patient visits for ILI are weighted on the basis of state population. Use of the national baseline for regional data is not appropriate.

§§ Activity levels are based on the percentage of outpatient visits in a jurisdiction attributed to ILI and are compared with the average percentage of ILI visits that occur during weeks with little or no influenza virus circulation. Activity levels range from minimal, which would correspond to ILI activity from outpatient clinics below or only slightly above the average, to high, which would correspond to ILI activity from outpatient clinics that are much higher than the average. Because the clinical definition of ILI is nonspecific, not all ILI is caused by influenza; however, when combined with laboratory data, the information on ILI activity provides a clearer picture of influenza activity in the United States.

¶¶ Levels of activity are 1) *no activity;* 2) *sporadic:* isolated laboratory-confirmed influenza case(s) or a laboratory-confirmed outbreak in one institution, with no increase in activity; 3) *local:* increased ILI, or at least two institutional outbreaks (ILI or laboratory-confirmed influenza) in one region of the state, with recent laboratory evidence of influenza in that region and virus activity no greater than sporadic in other regions; 4) *regional:* increased ILI activity or institutional outbreaks (ILI or laboratory-confirmed influenza) in at least two but less than half of the regions in the state with recent laboratory evidence of influenza in those regions; and 5) *widespread:* increased ILI activity or institutional outbreaks (ILI or laboratory-confirmed influenza) in at least half the regions in the state, with recent laboratory evidence of influenza in the state.

*** FluSurv-NET conducts population-based surveillance for laboratory-confirmed influenza-associated hospitalizations among children aged <18 years (since the 2003–04 influenza season) and adults aged ≥18 years (since the 2005–06 influenza season). FluSurv-NET covers approximately 70 counties in the 10 Emerging Infections Program states (California, Colorado, Connecticut, Georgia, Maryland, Minnesota, New Mexico, New York, Oregon, and Tennessee) and additional Influenza Hospitalization Surveillance Project (IHSP) states. IHSP began during the 2009–10 season to enhance surveillance during the 2009 H1N1 pandemic. IHSP sites included Iowa, Idaho, Michigan, Oklahoma, and South Dakota during the 2009–10 season; Idaho, Michigan, Ohio, Oklahoma, Rhode Island, and Utah during the 2010–11 season; Michigan, Ohio, Rhode Island, and Utah during the 2011–12 season; Iowa, Michigan, Ohio, Rhode Island, and Utah during the 2012–13 season; and Michigan, Ohio, and Utah during the 2013–14 season.

††† Incidence rates are calculated using CDC’s National Center for Health Statistics population estimates for the counties included in the surveillance catchment area. Laboratory confirmation is dependent on clinician-ordered influenza testing, and testing for influenza often is underused because of the poor reliability of rapid influenza diagnostic test results and greater reliance on clinical diagnosis for influenza. As a consequence, the number of cases identified as part of influenza hospitalization surveillance likely is an underestimation of the actual number of persons hospitalized with influenza.

§§§ The seasonal baseline proportion of P&I deaths is projected using a robust regression procedure, in which a periodic regression model is applied to the observed percentage of deaths from P&I that were reported by the 122 Cities Mortality Reporting System during the preceding 5 years. The epidemic threshold is set at 1.645 standard deviations above the seasonal baseline.
